# The dawn of a new era: can machine learning and large language models reshape QSP modeling?

**DOI:** 10.1007/s10928-025-09984-5

**Published:** 2025-06-16

**Authors:** Ioannis P. Androulakis, Lourdes Cucurull-Sanchez, Anna Kondic, Krina Mehta, Cesar Pichardo, Meghan Pryor, Marissa Renardy

**Affiliations:** 1 Biomedical Engineering, Rutgers University, Piscataway, NJ USA; 2 Pharmetheus, AB, Uppsala, Sweden; 3 Bristol-Myers Squibb Company, Princeton, NJ USA; 4 Kyowa Kirin, Princeton, NJ USA; 5 AstraZeneca R&D, Systems Medicine, Cambridge, CB2 0AA UK; 6 Johnson & Johnson Innovative Medicine, Spring House, PA USA; 7 GSK, Collegeville, PA USA; 8 Quantitative Systems Special Interest Group (QSP SIG), International Society of Pharmacometrics (ISoP), Bridgewater, USA

**Keywords:** Quantitative Systems Pharmacology (QSP), Machine Learning (ML), Artificial Intelligence (AI), Large Language Models (LLMs), Drug Development, Hybrid Modeling, Model-Informed Drug Development (MIDD)

## Abstract

Quantitative Systems Pharmacology (QSP) has emerged as a cornerstone of modern drug development, providing a robust framework to integrate data from preclinical and clinical studies, enhance decision-making, and optimize therapeutic strategies. By modeling biological systems and drug interactions, QSP enables predictions of outcomes, optimization of dosing regimens, and personalized medicine applications. Recent advancements in artificial intelligence (AI) and machine learning (ML) hold the potential to significantly transform QSP by enabling enhanced data extraction, fostering the development of hybrid mechanistic ML models, and supporting the introduction of surrogate models and digital twins. This manuscript explores the transformative role of AI and ML in reshaping QSP modeling workflows. AI/ML tools now enable automated literature mining, the generation of dynamic models from data, and the creation of hybrid frameworks that blend mechanistic insights with data-driven approaches. Large Language Models (LLMs) further revolutionize the field by transitioning AI/ML from merely a tool to becoming an active partner in QSP modeling. By facilitating interdisciplinary collaboration, lowering barriers to entry, and democratizing QSP workflows, LLMs empower researchers without deep coding expertise to engage in complex modeling tasks. Additionally, the integration of Artificial General Intelligence (AGI) holds the potential to autonomously propose, refine, and validate models, further accelerating innovation across multiscale biological processes. Key challenges remain in integrating AI/ML into QSP workflows, particularly in ensuring rigorous validation pipelines, addressing ethical considerations, and establishing robust regulatory frameworks to address the reliability and reproducibility of AI-assisted models. Moreover, the complexity of multiscale biological integration, effective data management, and fostering interdisciplinary collaboration present ongoing hurdles. Despite these challenges, the potential of AI/ML to enhance hybrid model development, improve model interpretability, and democratize QSP modeling offers an exciting opportunity to revolutionize drug development and therapeutic innovation. This work highlights a pathway toward a transformative era for QSP, leveraging advancements in AI and ML to address these challenges and drive innovation in the field.

## Introduction

Quantitative Systems Pharmacology (QSP) holds significant promise in revolutionizing drug development and therapeutic innovation [1–3]. By integrating mathematical modeling, computational simulations, and systems biology, it serves as an integrative knowledge framework, especially for complex diseases. QSP provides mathematical models of biological systems that can be used to generate hypotheses, identify critical system parameters, and simulate scenarios that are otherwise difficult to test. This approach enables a mechanistic understanding of complex biological processes and drug interactions [4], allowing researchers to optimize drug dosing regimens and assess drug efficacy and safety earlier in development. QSP offers several key benefits: improved decision-making by helping to identify optimal drug targets, more efficient clinical trial designs, and risk reduction of clinical failures by simulating various scenarios before real-world testing. QSP also has the potential to provide personalized medicine by modeling patient variability, enabling the tailoring of therapies to specific patient subgroups, enhancing treatment effectiveness, and minimizing adverse effects.

The promise of QSP lies in its ability to provide a robust framework for integrating data from preclinical and clinical studies, improving drug development efficiency, enhancing patient outcomes, and paving the way for innovative, safer, and more effective treatments [5–9]. Its potential to reduce development costs and timelines while increasing the probability of success makes it a cornerstone of modern drug development strategies. The framework’s versatility gives rise to models of varying complexity spanning the spectrum from detailed ODE representations [10] to complex computational methodologies [11]. QSP empowers researchers to explore various data modalities and computational approaches. This flexibility has led to the realization that building a QSP model, unlike a more typical pharmacometrics model, requires that scientists focus extensively on key steps of model development [7]. These, broadly, include: (1) defining appropriate needs statements; (2) reviewing relevant biological, physiological, and clinical data; (3) identifying appropriate representations of biological, physiological, and clinical knowledge; (4) determining appropriate methodologies for capturing expected behaviors and systematically assessing the model predictions; and finally, (5) appropriately use the model, and the associated analyses, to generate testable hypotheses.

The growing number of QSP-informed submissions to regulatory agencies highlights its increasing influence, demonstrating credibility in guiding dose selection, dosing regimens, and risk mitigation strategies [12]. By predicting potential risks and outcomes early, QSP ensures cost and time efficiency by reducing the need for costly and time-consuming trial-and-error experiments, thereby accelerating the development timeline. Furthermore, QSP’s versatility allows it to address challenges across diverse therapeutic areas, including hematology, oncology, rare diseases, and pediatric drug development. As highlighted in a recent report, QSP has emerged as an increasingly valuable tool in drug development, evidenced by the growing number of submissions to the U.S. FDA over the past decade [13]. Its applications include dose selection, optimization of dosing regimens, and providing insights for clinical trials, particularly for rare diseases [14], focusing on drug efficacy and safety. QSP submissions highlight its versatile use cases while supporting the design and optimization of clinical trials for adult and pediatric populations.

In developing a QSP model, the choices made regarding the nature of the mathematical (or computational) model are critical. Given the uncertainty that often surrounds the development of mechanistic QSP models, it is therefore important to appreciate that in QSP, not only are the model predictions a key deliverable, but also the model itself, including the process of developing the model. Since the early days of QSP model development, primary emphasis has been placed on critical methodological aspects, focusing on model structure, parameter quantification, and assessment [15, 16]. It should come as no surprise that recent developments in artificial intelligence (AI) and machine learning (ML) were quickly recognized as major opportunities for further advancing QSP [17, 18].

**Fig. 1:**
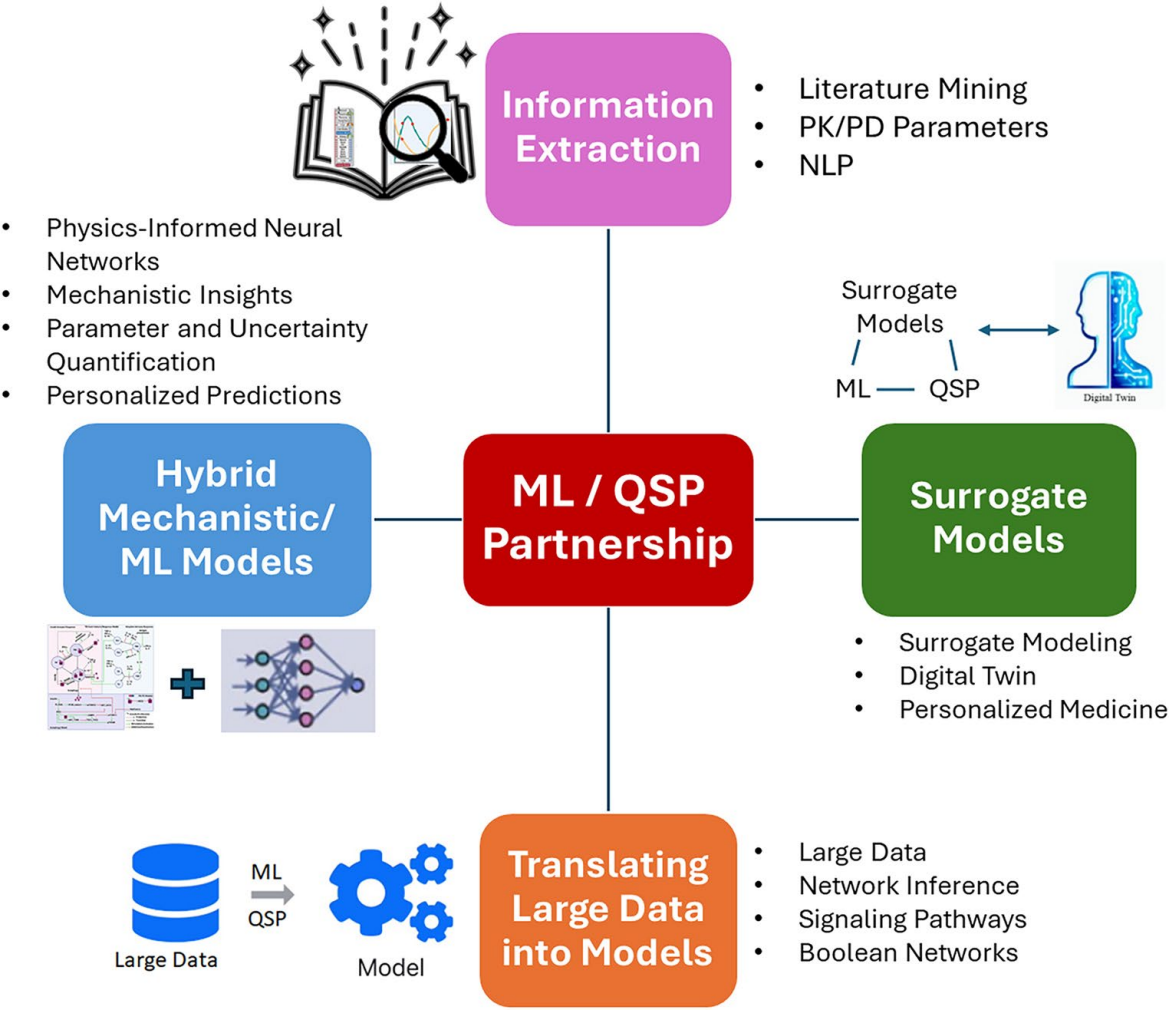
Integration of machine learning and quantitative systems pharmacology: leveraging information extraction, hybrid mechanistic/ML models, surrogate models, and data translation to enhance model development and enable digital twin applications

In this paper, we wish to explore how AI/ML might reshape the mathematical/computational model development process in QSP. In doing so, we will explore the ability of AI/ML to assist in four primary tasks: (1) extracting information, (2) developing surrogate models, (3) translating data into models, and (4) developing hybrid mechanistic/ML models. Finally, we will address whether AI/ML will eventually transition from a tool to a partner, Fig. 1. In doing so, we will not provide any further definitions of QSP or AI/ML and will only state that “[…] *AI is the broader concept of enabling a machine or system to sense*,* reason*,* act*,* or adapt like a human; ML is an application of AI that allows machines to extract knowledge from data and learn from it autonomously*^1^.”

## Using ML to extract and represent information during the model development process

Extracting and representing relevant information is foundational in developing mathematical models, ensuring accuracy, reliability, and utility [19]. This process involves identifying and organizing key data, ensuring traceability throughout the data lifecycle, translating it into mathematical or computational structures that reflect the biological, chemical, or physical systems under investigation, and defining the model’s scope and objectives. Effective extraction clarifies the questions the model seeks to answer, avoids unnecessary complexity, and ensures essential components are included. Researchers can design scientifically robust frameworks tailored to specific study needs by grounding the model in comprehensive and well-curated information. An efficient extraction process leverages existing experimental findings, prior models, and validated knowledge, reducing redundancy while saving time and resources. It also provides a structured basis for parameter estimation, calibration, and validation, ensuring that observed data, rather than hypothetical assumptions, guide the model. Knowledge gaps often become evident during this process, highlighting areas for additional experiments or analyses to improve the model’s completeness and accuracy.

Organizing information in a transparent and reproducible format fosters collaboration among interdisciplinary teams, allowing diverse expertise to contribute to model development. This transparency also helps ensure regulatory compliance by justifying model assumptions and data sources required by regulatory authorities, such as the US FDA or the European Medicine Agency (EMA). Additionally, well-represented information ensures model flexibility and scalability, enabling the integration of patient variability to support personalized medicine or refine dosing regimens for complex diseases. Ultimately, this step aligns the model with real-world systems, facilitates collaboration, and enables regulatory acceptance, forming the cornerstone of effective model development. PKPD parameters define drug absorption, distribution, metabolism, elimination, and target interactions and serve as the backbone for simulating drug behavior and its effects within biological systems. Extracting PKPD values from literature ensures the model is built on validated, experimentally derived data, enhancing credibility and reducing the need for redundant studies. Accurate values are indispensable for predicting drug efficacy and safety across diverse scenarios, enabling the development of robust and reliable models.

Literature mining is essential to this endeavor, systematically gathering and synthesizing knowledge from vast amounts of published data [20]. This step ensures models are grounded in validated findings and incorporate relevant biological, chemical, and pharmacological insights. AI and ML advances have significantly enhanced literature mining by automating data identification, extraction, and categorization from diverse sources [21]. AI/ML tools efficiently analyze complex datasets, detect patterns, and prioritize relevant content, uncovering connections often overlooked by manual methods. These capabilities accelerate the process and improve the accuracy and depth of extracted knowledge. The ability of AI/ML to process and integrate large-scale data has transformed literature mining into an indispensable component of model development, providing a robust foundation for predictive modeling in fields like QSP. However, a notable concern is that the quality of data mining can vary significantly depending on the topic, potentially impacting the reproducibility and reliability of results, especially in complex fields like biomedical research [22].

Early efforts [23] demonstrated the feasibility of literature mining for retrieving PK numerical data. Such approaches addressed the difficulties associated with the manual curation of thousands of literature entries. Alternatively, literature mining “trains” a system to discover helpful information or make reasonable hypotheses based on available publications. Early efforts focused on entity recognition, information extraction, and outlier detection. Later approaches recognized the need to develop additional standards, i.e., ontologies, for proper annotation and effective searching [24]. Most recent efforts have been to create comprehensive ML pipelines by developing appropriate annotations that maximize the classification capabilities of the search tools [25]. Such approaches offer the possibility of effectively mining hundreds of thousands of documents. Leveraging advances in natural language processing (NLP) [26] demonstrated a workflow showcasing the expedited extraction and analysis of large amounts of information from regulatory review documents.

Recent approaches further illustrate how applying ML and mechanistic modeling can accomplish preclinical exposure prediction [27]. ML learning establishes relations between molecular structures and PK parameters, which are then used as input to a secondary ML layer that predicts exposure. In [28], it was shown how ML could learn structure-activity relationships, which were used to predict PK of untested compounds. A similar approach was also proposed by [29], showing how ML tools obtain time-dependent PK and ADME parameters, which are then used to develop predictive models incorporated into more complex mechanistic models.

In short, ML has the potential to enable QSP scientists to streamline the literature landscape and provide a comprehensive framework for systematizing predictions. In other words, in one shot, one can now go from surveying the literature to making predictions that can be used as foundational steps for further model development.

## Using ML to translate data to dynamic models

Knowing the components and their interactions is essential for developing mechanistic QSP models, as they provide the foundational framework for the model’s structure. Components represent biological entities, such as proteins, genes, cells, or tissues. At the same time, interactions capture the dynamic relationships between these entities, such as biochemical reactions, signaling pathways, and pharmacological or physiological processes. A comprehensive understanding of these aspects ensures that the model better reflects the underlying biology of the system, as QSP models focus on integrating mechanistic details across multiple biological scales to simulate how a drug affects a system and how the system responds.

System structure in QSP models is typically developed based on prior knowledge derived from established literature, experimental data, and expert insights [9]. This process involves explicitly identifying and validating the components and interactions within the biological system to ensure that the model captures the known mechanisms accurately. Researchers may rely on subject matter experts and curated databases of protein-protein interactions, signaling pathways, or pharmacokinetic and pharmacodynamic relationships to outline the framework of the system. However, while this approach ensures that the model aligns with established scientific understanding, it is often limited by the availability and scope of existing knowledge, which may overlook novel or context-specific interactions. Realizations based on manual curation tend to reflect small-scale causal relationships.

Machine learning complements this traditional process by enabling the interpretation of large-scale, complex datasets to uncover previously unrecognized components and interactions. It proves particularly useful in cases with multiple variables with very few observations vs. a clinical dataset with few and many observations. Unlike manual curation, ML can identify patterns, correlations, and dependencies within high-dimensional data, offering a data-driven perspective that can augment and refine the system structure. For instance, ML techniques such as network inference [30] can predict regulatory or signaling relationships based on gene expression or proteomics data. At the same time, clustering algorithms can reveal emergent substructures or functional modules. By integrating these insights with prior knowledge, researchers can expand and adapt the QSP model’s framework to reflect established and novel biological mechanisms, increasing its accuracy and predictive power. This dynamic interplay between expert-driven and data-driven approaches ensures that QSP models remain robust and relevant in capturing the complexity of biological systems and their responses to therapeutic interventions. Broadly, one can identify two likely areas where ML plays a significant role, depending on how data is treated and incorporated: network inference and direct translation of data into models.

Network inference has been a foundational concept in systems biology [31]. It refers to the computational process of reconstructing interaction networks from biological data to understand the relationships between various components, such as genes, proteins, or metabolites. By analyzing high-dimensional datasets, such as transcriptomics or proteomics, network inference algorithms identify dependencies, correlations, or causal interactions representing biological processes like signaling pathways, regulatory networks, or metabolic systems. Machine learning has significantly developed such approaches [32]. As illustrated nicely by [33], ML can assist in providing, using omics datasets, predictions of new interactions between genes and proteins that can be integrated within prior QSP mechanistic models. Such pipelines offer an excellent starting point for merging extensive databases with large-scale mechanistic models.

Secondly, recent approaches examined the possibility of converting data hypothesis-neutral into dynamic QSP models. Researchers in [34] present a systematic pipeline for converting biological data into dynamic QSP models through Boolean network inference [35]. The process begins with data collection and binarization, where continuous data are converted into binary states using methods like k-means clustering. These binary states represent biological species’ “on” and “off” activity levels. A Boolean network is then constructed by inferring logical relationships between components, employing techniques such as the Best-Fit Extension method. The resulting Boolean network provides a qualitative representation of system interactions and can undergo pruning and sensitivity analysis to refine and simplify its structure. Subsequently, the Boolean network is transformed into a dynamic model using ordinary differential equations (ODEs), leveraging homologs of Boolean functions to approximate continuous behaviors [36]. A similar approach was also demonstrated in [37] using a generalized model approximation [38]. Converting data into models is quite powerful as it shows that the essential elements of a QSP building process can, in principle, be automated, provided that appropriate data exists.

## Combining QSP, surrogate models, and ML to build digital twins

Surrogate modeling and digital twins are closely related concepts that often complement each other, particularly in complex systems modeling and simulation [39]. A surrogate model is a simplified mathematical or computational representation of a more complex system designed to approximate its behavior while significantly reducing the computational cost. These models are typically derived from data generated by high-fidelity simulations or experiments. They are used to accelerate analyses, optimize designs, or explore otherwise computationally prohibitive scenarios. In contrast, a digital twin is a dynamic, virtual representation of a physical system or process that evolves, integrating real-time data to provide insights, monitor performance, and predict outcomes. The relationship between these two lies in their synergistic roles within a modeling ecosystem. Surrogate models are often embedded within digital twins to enhance their efficiency, enabling the twin to perform real-time predictions or iterative simulations without relying on the full computational complexity of the original system. Related to surrogate models are virtual populations consisting of a simulated representation of a group of subjects, mimicking a real-world population’s characteristics, behaviors, or interactions. In quantitative systems pharmacology (QSP) and drug development, virtual populations are used to simulate diverse patient responses, enabling the prediction of drug efficacy, safety, and optimal dosing in different subpopulations [40–42].

A digital twin of a drug development pipeline might use surrogate models to quickly simulate pharmacokinetic and pharmacodynamic (PKPD) outcomes across a range of dosing strategies [43–46]. This integration allows the digital twin to remain adaptive and responsive to new data while retaining the predictive power of the underlying high-fidelity models. Moreover, the data streams and insights generated by digital twins can inform the development of surrogate models. As digital twins continually ingest real-time data and refine their predictions, they can provide updated or enriched datasets that enhance the accuracy and relevance of the surrogate models. This feedback loop creates a robust framework where surrogate models and digital twins iteratively improve each other, enabling more robust decision-making, optimization, and scenario analysis. In essence, surrogate models provide the computational efficiency for digital twins to operate in real-time or near-real-time settings. In contrast, digital twins offer a dynamic and data-driven environment for deploying and refining surrogate models. Together, they form a complementary relationship that enables the simulation, monitoring, and optimization of complex systems in practical and innovative ways.

Machine learning plays a pivotal role in enabling the development of digital twins in quantitative systems pharmacology by integrating patient-specific data with mechanistic models to create highly personalized simulations of drug behavior and therapeutic outcomes [47]. QSP models can generate high-fidelity, simulation-based datasets that capture various physiological and pharmacological scenarios, including edge cases or rare conditions. These datasets can be used to train ML-based surrogates, which mimic the behavior of the full QSP model while being computationally efficient. By learning from both real-time and historical patient data, as well as QSP-simulated data, ML enhances the ability of digital twins to dynamically adapt and predict responses to different dosing regimens or therapeutic interventions. This synergy between ML and QSP supports the creation of robust digital twins and facilitates the modeling of patient variability, optimization of treatment strategies, and advancements in personalized medicine [40, 48, 49].

## Hybrid ML-mechanistic models

While firmly rooted in established scientific principles, mechanistic models often face scalability, parameterization, and uncertainty quantification challenges, mainly when incomplete system knowledge or datasets are large and complex [50, 51]. Conversely, machine learning models are adept at identifying patterns and making predictions from vast datasets but often lack interpretability, biological plausibility, and the ability to generalize reliably beyond the training data. The integration of these approaches has emerged to bridge these gaps, enabling the construction of models that are both more interpretable and capable of leveraging data-driven insights to uncover novel relationships, refine parameter estimates, and make robust predictions. This need became especially acute in systems biology and pharmacology, where dynamic interactions across multiple scales and heterogeneous data sources are common. Yet, comprehensive mechanistic knowledge is often unavailable or incomplete.

Hybrid, that is, mechanistic + machine learning, models offer a powerful alternative for tackling complex systems where complete mechanistic understanding is elusive. By integrating established mechanistic principles with data-driven machine learning, these models can leverage the strengths of both approaches. Specifically, they allow us to retain mechanistic descriptions for well-understood system parts while employing ML components to capture relationships that are too complex or poorly understood to be modeled mechanistically [52]. ML components can fill knowledge gaps, parameterize models, or infer relationships not captured by existing mechanistic theories, allowing for greater adaptability in dynamic or poorly understood systems. This will enable us to effectively represent and extract insights from system components that defy mechanistic explanation but exhibit quantifiable patterns within data, allowing a more complete and accurate representation of the overall system. This approach provides a pragmatic way to build predictive and insightful models, even when faced with knowledge gaps, by using data to inform and enhance our understanding where explicit mechanisms are unknown. Therefore, the development of hybrid mechanistic/ML models has been driven by the need to address the limitations of purely mechanistic or purely data-driven approaches in understanding and predicting the behavior of complex biological systems [53]. This integration can be performed by using physics-informed machine learning or a hybrid modeling approach:

### Physics-informed machine learning (PIML)

In this approach, mechanistic insights are explicitly incorporated into the ML algorithms to guide or constrain the learning process [54]. The integration might include embedding known equations, boundary conditions, or conservation laws into the ML model architecture or the loss function. This method ensures that the ML model adheres to the physical or mechanistic constraints of the system, improving generalization and interpretability while reducing the reliance on large datasets. The work in [55] demonstrated the potential of a Physics-Informed Neural Network (PINN) and fractional Physics-Informed Neural Networks (fPINNs), integrating ODEs with integer/fractional derivative order from compartmental modeling with neural networks to improve prediction of complex drug responses. A PINN in a QSP context is a hybrid modeling framework that combines the interpretability of mechanistic models with the flexibility of machine learning. In this approach, the neural network learns to approximate system behavior, such as drug concentration-time profiles or therapeutic effects, while being constrained by the underlying mathematical principles governing the system, such as ODEs or partial differential equations describing absorption, distribution, metabolism, and excretion (ADME) or drug-target interactions. The PINN leverages observed data and embedded physics to estimate key pharmacological parameters and predict system dynamics simultaneously. The physics-informed loss function ensures that the network’s predictions adhere to known biological and physiological laws, improving robustness and generalizability even with sparse or incomplete datasets. This enables PINNs to capture the complexity of QSP systems, infer latent dynamics, and provide biologically interpretable insights, bridging the gap between traditional mechanistic models and data-driven approaches.

### Composite models combining mechanistic and ML models

Different parts of the problem are modeled using distinct techniques based on their characteristics or data availability. For example, components of the system that are well-understood and governed by first principles are modeled mechanistically. In contrast, poorly understood or data-rich elements are modeled using ML [52]. Mechanistic models rely on well-defined, directly measurable, expert variables such as enzyme concentrations, receptor occupancy, or drug pharmacokinetics. Grounded in first principles and experimental evidence, these variables provide interpretable insights into fundamental biological processes and are typically derived from controlled laboratory experiments. However, many of these variables become unobservable or impractical to measure in clinical settings. For instance, while a laboratory might precisely track drug-receptor binding affinity, clinical measurements often focus on aggregate outcomes like efficacy or biomarkers. This limitation poses challenges for purely mechanistic models, which struggle to capture real-world variability and systemic interactions. By contrast, ML models thrive on observable variables like blood pressure, genetic profiles, or patient demographics, which are directly measured in clinical settings. However, their lack of biological interpretability often results in black-box predictions. Hybrid models combine these strengths, using mechanistic components to model biologically defined variables and anchor the system in established knowledge.

In contrast, ML components handle data-rich, complex, or less mechanistically understood variables, such as clinical scores, patient variability, or biomarker interactions. The mechanistic model might describe pharmacokinetics in a hybrid approach, while ML predicts patient-specific outcomes based on mechanistic variables and additional demographic or genomic factors. This division of labor balances interpretability with flexibility, ensuring hybrid models remain grounded in evidence while scaling to diverse real-world data. The progression from mechanistic models to ML models and, ultimately, hybrid models highlight the complementary nature of these approaches, particularly when it comes to the types of variables each method can effectively model [56, 57]. In [58], for example, it was demonstrated how a hybrid, mechanistic + neural network model could be built so that a neural network absorption model is also learned while estimating the mechanistic model parameters. Meanwhile, in [59], a hybrid model that can enable personalized survival prediction was demonstrated.

## Large Language models: transitioning AI/ML from “tool” to “partner”

It has become evident that ML plays a central role in QSP, offering a unique opportunity to incorporate advances in data science to boost its impact [60]. As our discussion has indicated, AI/ML is treated as a sophisticated toolbox that assists in computationally intensive tasks and supports existing methodologies that have withstood the test of time [61]. Treating AI/ML as a toolbox must be held to standards at least as high as those of conventional model assessment. However, studies have highlighted the need for caution, suggesting that AI/ML should not be unquestioningly trusted without critical evaluation [62]. This is a valid criticism, and careful assessment processes must be implemented before turning away all AI/ML tools.

In contrast to the traditional use of ML solely as a tool, the emergence of ChatGPT^2^ (https://openai.com*)* has marked a pivotal moment, dramatically shifting the landscape of AI/ML by introducing broadly Large Language Models (LLMs) as transformative tools. LLMs are advanced AI systems trained on vast amounts of text data to understand and generate human-like language [63]. They use deep learning architectures to process and analyze text, enabling them to perform tasks like summarizing information, answering questions, writing code, and translating languages. LLMs are designed for conversational interactions, offering users coherent, context-aware responses. They have already been widely used in various fields, including education, healthcare, and research, for their ability to simplify complex concepts, assist with problem-solving, and enhance productivity through natural language understanding and generation [64].

Various large language models (LLMs) are currently available, each offering unique capabilities suited to different applications. OpenAI’s ChatGPT (https://chatgpt.com), powered by the GPT-4 architecture, is widely used for conversational AI, coding assistance, and creative content generation. Google DeepMind’s Gemini (https://gemini.google.com/app) integrates advanced reasoning capabilities with language understanding, reflecting Google’s latest efforts in general-purpose AI. Anthropic’s Claude (https://claude.ai/new) emphasizes safety and constitutional AI, making it suitable for aligned and controllable deployment in sensitive contexts. Meta’s LLaMA (Large Language Model Meta AI) is designed primarily for academic research, offering open-access models to support foundational exploration in language modeling. From the open-source community, Hugging Face’s BLOOM (https://huggingface.co/bigscience/bloom) is a multilingual model developed collaboratively by the BigScience initiative, while Falcon (https://falconllm.tii.ae) developed by the Technology Innovation Institute (TII) in the UAE provides high-performance, openly available LLMs suitable for fine-tuning and deployment. Finally, Cohere’s Command (https://cohere.com/command) focuses on enterprise and developer use cases, particularly in generating business-specific, technical, and structured content. DeepSeek’s open-source models, including DeepSeek (https://www.deepseek.com/ ), offers high-performance alternatives for both code generation and general-purpose reasoning. Their transparent release strategy and multilingual training corpus make them valuable tools for research and developer applications.Together, these LLMs span the spectrum from open research to commercial deployment, providing a robust toolkit for advancing AI across disciplines. Beyond the dominant offerings from OpenAI, Google DeepMind, and Anthropic, a rapidly expanding ecosystem of regional and open-source LLMs is reshaping the AI landscape. Models like Baidu’s Ernie Bot, Naver’s HyperCLOVA X, Mistral’s efficient open models, and India’s OpenHathi underscore a global shift toward sovereign and multilingual AI development. Meanwhile, specialized models such as Code Llama, Command R+, and WizardLM demonstrate the growing demand for tailored solutions in coding, retrieval-augmented generation, and instruction tuning. As this diversification accelerates, it promises to democratize AI access, foster innovation through competition, and challenge the hegemony of a few centralized players—setting the stage for a more dynamic and inclusive future for LLMs.

Even more exciting is that several platforms that leverage LLMs for specialized niche areas have emerged. A (small) sample includes PubMedGPT (https://chatgpt.com/g/g-NOGyHbsFG-pubmed-gpt ), which aids researchers in summarizing and analyzing biomedical literature; BioGPT (https://huggingface.co/docs/transformers/en/model_doc/biogpt ), designed to generate insights from biomedical data for tasks such as gene-disease associations; BloombergGPT [65], which specializes in financial analysis, market trends, and news summarization; Khanmigo (https://www.khanmigo.ai/ ), an AI tutor offering personalized learning support for students and educators; WolframAlpha Pro (https://www.wolframalpha.com/pro), which combines computational tools with LLMs for scientific problem-solving; GitHub Copilot (https://github.com/features/copilot ), a programming assistant that suggests code snippets and helps developers debug in real time; Ada Health (https://ada.com/ ), which provides personalized health assessments and care recommendations; PolicyGPT [66], designed to analyze and draft policy documents with precision.

As LLMs become increasingly integrated into complex tasks, the need for efficient and precise prompts has become essential, giving rise to the discipline of prompt engineering [67]. A prompt refers to the input or instruction provided to an LLM to elicit a desired response, making its design crucial for achieving accurate and context-relevant outputs. This emerging field focuses on crafting effective prompts to optimize the performance of LLMs. By designing precise, context-aware instructions, prompt engineering enables users to harness LLM capabilities for specific tasks, including computational and mathematical modeling. In these domains, well-constructed prompts can guide LLMs to generate accurate code for numerical solvers, translate biological or physical systems into mathematical representations, and debug or refine existing models. This practice bridges the gap between human expertise and AI capabilities, ensuring that LLM outputs align with domain-specific requirements. As such, prompt engineering is pivotal in leveraging LLMs as collaborative tools, enhancing their utility in solving complex modeling problems efficiently and accurately.

Prompt engineering is a relatively new field within artificial intelligence that focuses on crafting the most effective input prompts to guide LLMs, producing the best possible outputs for specific tasks, essentially acting as a way to optimize how you communicate with AI systems to get the desired results; it is considered a critical skill as the capabilities of these models continue to develop. A prompt engineer must understand how LLMs work and natural language processing (NLP) techniques and craft well-structured prompts that effectively convey the desired task and context. With the increasing adoption of LLMs, prompt engineering is becoming recognized, demanding expertise in prompt design and optimization^3^.

Scientists are already exploring the possibilities of conversing with an LLM to obtain parameters from the literature and generate computer code implementing pharmacometrics models of interest [68, 69]. The impact of prompt strategies to generate code, perform calculations, and generate scientific text in a PK context was further examined in [70], whereas the ability of two LLMs to generate relevant NONMEM code was assessed in [71]. Most recently, researchers have begun exploring the provocative idea of using LLMs not just as information retrieval tools, but as neutral mediators in scientific debates, including those surrounding the evolving role of AI/ML in Quantitative Systems Pharmacology (QSP). In this context, LLMs were tested for their ability to distill opposing viewpoints, identify points of convergence, and propose balanced frameworks, highlighting their potential to arbitrate complex methodological tensions, such as how AI/ML should complement mechanistic modeling in QSP [72]. The critical thing to remember is that in these applications, the interactions with the LLMs were made using generic (agnostic) approaches and language using lay terms. Thus, the pivotal change is that the user does not need to be a domain “expert”! One can identify many issues associated with accuracy and correctness, but we must also realize that we only witness the early beginnings of an upcoming revolution.

## So, what’s next

The arrival of large language models has the potential to significantly transform the landscape of computational modeling, with profound implications across disciplines [73]. QSP will not be an exception. LLMs bridge knowledge gaps, enhance collaboration, and democratize the creation and consumption of scientific knowledge [74]. By acting as intelligent assistants, LLMs enable researchers without deep coding or domain expertise to engage with advanced modeling workflows. They assist in generating code for numerical solvers, translating biological problems into mathematical representations, and debugging or optimizing complex pipelines. This reduces barriers to entry, allowing a broader range of individuals to contribute to QSP advancements. Early comparisons already indicate that LLMs have the potential to serve as reliable programming assistants [75, 76].

One of the most impactful contributions of LLMs will be their ability to accelerate hypothesis testing by facilitating rapid iteration cycles in model development, suggesting mathematical frameworks, and generating scripts for simulating complex systems. In parallel, LLMs enable the contextual integration of biological knowledge, synthesizing insights from literature, automating parameter extraction, and bridging disparate datasets to inform model structures and assumptions. This is particularly valuable in QSP, where multiscale modeling combines molecular, cellular, and systemic dynamics. LLMs could streamline this integration by translating models between domains and suggesting modular frameworks to integrate diverse biological processes. LLMs also enhance the handling of data, a critical aspect of ML-driven modeling. They automate preprocessing steps such as feature selection, outlier detection, and scaling, making it easier to manage high-dimensional datasets like those from omics studies or clinical trials.

Additionally, they could contribute to improved model interpretability by summarizing structures, assumptions, and results in natural language, creating clear documentation, and translating technical outputs, such as stability analyses, into actionable insights. The seamless integration of ML with mechanistic models could become a foundational aspect of QSP, and LLMs will play a pivotal role here. They could assist in designing hybrid frameworks that combine mechanistic principles with data-driven components, such as neural networks while automating hyperparameter tuning and validation. This synergy between approaches enhances the scalability of QSP efforts, enabling collaborative work among interdisciplinary teams. LLMs serve as a shared interface, aligning objectives and assisting with high-performance computing, cloud integration, and model deployment.

LLMs will empower quantitatively trained professionals, such as mathematicians, physicists, engineers, and data scientists, to engage with life scientists and clinicians. By providing real-time explanations of biological concepts, decoding specialized terminology, and offering contextual insights, LLMs reduce the steep learning curve often associated with transitioning into life sciences. They facilitate cross-disciplinary communication, enabling experts in mathematical modeling to translate their work into biologically meaningful terms and fostering collaboration with pharmacologists and biologists. Interactive learning pathways supported by LLMs accelerate onboarding, suggesting resources, generating example problems, and automating code generation for biological simulations. LLMs also streamline the development of mathematical models in biology and pharmacology by assisting in parameter identification, translating biological systems into mathematical frameworks, and enabling multiscale integration. They simplify literature review and insight extraction, summarizing papers, extracting experimental findings, and synthesizing raw data into actionable insights. This capability promotes innovation by allowing researchers to safely explore biological questions, propose novel hypotheses, and rapidly develop computational prototype models.

In summary, LLMs will drive the evolution of machine learning from a tool whose use requires a significant level of expertise to an active partner in mathematical and computational modeling in QSP. By integrating natural language understanding with advanced computational capabilities, LLMs assist researchers in automating data extraction, code generation, and parameter optimization, facilitating interdisciplinary collaboration and hypothesis generation. Their ability to contextualize biological and mathematical knowledge enables seamless translation between data, models, and real-world applications. LLMs will reshape QSP by reducing barriers, enhancing collaboration, and enabling faster, more accessible model development. They bridge the gap between quantitative and biological domains, democratizing access to pharmacology and biology for quantitatively trained professionals while providing biological scientists access to computational methods and AI/ML, eventually accelerating innovation in systems pharmacology. LLMs will foster a more integrated and collaborative approach to addressing complex drug discovery and personalized medicine challenges.

**Fig. 2:**
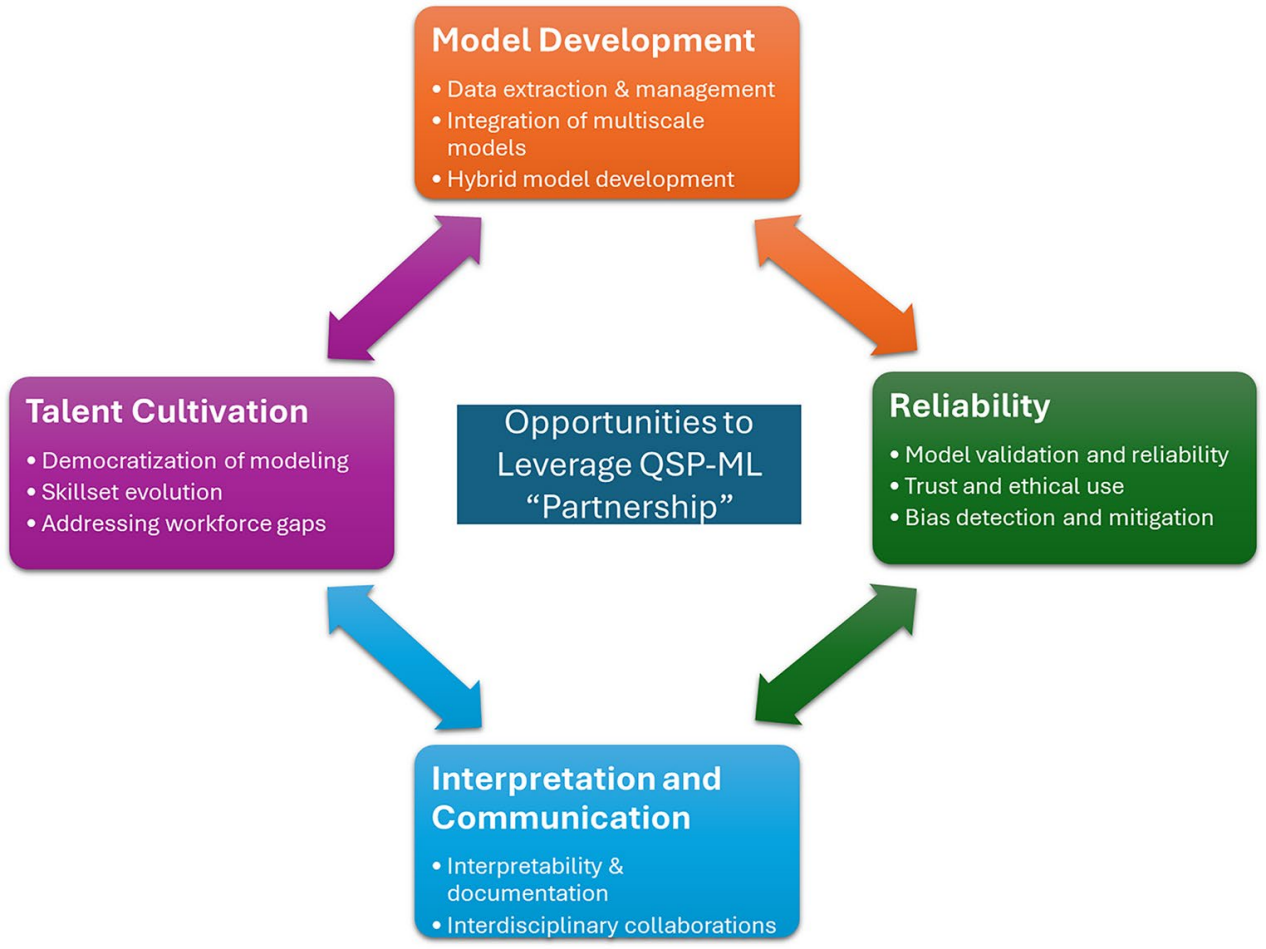
Opportunities to leverage QSP-ML partnership: advancing model development, ensuring reliability, enhancing interpretation and communication, and cultivating talent to address emerging challenges and drive innovation

Despite advancements, challenges remain. LLM-assisted QSP models must be rigorously validated; thus, regulatory oversight is critical [77]. LLM evaluation is already emerging as a crucial need [78], while domain-specific assessments are rising [79–82]. While LLMs simplify complex concepts, there is a risk of oversimplification or over-reliance, necessitating a fundamental understanding of the underlying science. Effective use of LLMs also requires fostering trust and collaboration between quantitative and biological experts to ensure interdisciplinary alignment, Fig. 2.

Nevertheless, magnificent challenges and opportunities await QSP in terms of workflow and workforce:

1. Model Validation, Reliability, Benchmarking, and Standardization: How can rigorous validation pipelines be established to ensure the accuracy and compliance of LLM-assisted models, especially in pharmacology, where safety and efficacy are paramount? What role can LLMs play in regulatory documentation and compliance assessments to streamline the approval process for QSP models? How can LLMs contribute to establishing benchmarks and standards for QSP models to ensure consistency and reproducibility across studies? Can they help harmonize frameworks used across industries or regions?

2. Integration of Multiscale Models: How can LLMs better assist in integrating multiscale biological processes, from molecular to systemic levels, while maintaining fidelity and reducing oversimplifications? Can LLMs help identify gaps or inconsistencies in multiscale models that might go unnoticed?

3. Data Management: What new approaches can LLMs introduce to automate the management of high-dimensional datasets, particularly in preprocessing, feature selection, and parameter extraction? How can LLMs enhance the integration of disparate datasets (e.g., omics and clinical trial data) into coherent QSP frameworks?

4. Hybrid Model Development: What best practices can emerge for combining mechanistic and data-driven components, such as neural networks, in hybrid frameworks designed by LLMs? How can LLMs address hyperparameter tuning and validation challenges to optimize the performance of hybrid QSP models?

5. Accelerating Hypothesis Generation and Experimental Design:

6. How can LLMs be leveraged to accelerate hypothesis generation and optimize experimental design in QSP research? Can they identify novel research questions, prioritize experiments based on predicted impact, or propose innovative study designs that maximize data utility? What strategies are needed to ensure LLM-generated hypotheses and designs are biologically plausible, scientifically rigorous, and aligned with regulatory requirements? How can these tools streamline the iterative cycle between modeling and experimentation?

7. Interpretability, Documentation, and Model Lifecycle Management: How can LLMs improve model interpretability by translating technical outputs into actionable insights for stakeholders? What methods can ensure that LLM-generated documentation (e.g., model assumptions and parameter choices) remains transparent and robust? What tools can LLMs provide for managing the lifecycle of QSP models, from development to retirement? How can they support version control, updates, and integration of new knowledge to maintain model relevance?

8. Interdisciplinary Collaboration: How can LLMs foster deeper trust and collaboration between quantitative and biological scientists to overcome cultural and disciplinary divides? Can LLMs act as unbiased mediators to align objectives and resolve misunderstandings in interdisciplinary projects?

9. Skillset Evolution: What new skillsets will researchers need to leverage LLMs effectively in QSP modeling? How can training programs and interactive learning pathways supported by LLMs accelerate onboarding experts from diverse disciplines into QSP?

10. Democratization^4^ of Modeling: How can LLMs lower barriers for professionals without deep domain or coding expertise to contribute meaningfully to QSP modeling? Could this democratization inadvertently lead to over-reliance on LLMs, and how can safeguards be implemented to ensure critical oversight by domain experts?

11. Addressing Workforce Gaps: Can LLMs help mitigate workforce shortages by enabling fewer individuals to accomplish more in QSP research? What strategies will ensure that LLM-driven automation augments rather than replaces the role of skilled professionals?

12. Trust and Ethical Use: How can the scientific community foster trust in LLM-assisted workflows, ensuring stakeholders view their outputs as reliable and ethical? What frameworks can ensure the responsible use of LLMs in QSP, particularly concerning data privacy, algorithmic bias, and reproducibility?

13. Artificial General Intelligence^5^ (AGI) in Mathematical/Computational Modeling and QSP: How could the advent of AGI revolutionize the development and application of mathematical models in QSP by achieving deeper, human-like comprehension of biological systems? Could AGI autonomously propose, refine, and validate models by simulating complex pharmacological scenarios across multiple scales? What ethical considerations emerge when delegating high-stakes decisions, such as model-based dosing strategies or risk-benefit analyses, to AGI systems, and how can safeguards be established to ensure accountability and interpretability?

14. Hallucinations and Scientific Accuracy: How can the QSP community guard against the risks posed by LLM hallucinations (plausible-sounding but incorrect or fabricated outputs [83]) in high-stakes modeling workflows? What mechanisms are needed to detect, flag, and correct hallucinated model assumptions, parameter values, or biological claims generated by LLMs? How can human-in-the-loop systems be designed to balance automation with expert oversight, especially when hallucinations may propagate errors into downstream simulations or regulatory documentation? Can techniques such as retrieval-augmented generation (RAG), grounded outputs, or model confidence estimation reduce hallucination risk in QSP applications?

One thing is certain: we have entered an exciting and, in many ways, unpredictable era!

## Data Availability

No datasets were generated or analysed during the current study.

## Footnotes

1 https://cloud.google.com/learn/artificial-intelligence-vs-machine-learning.

2 ChatGPT (Chat Generative Pre-trained Transformer) referring to an advanced conversational (i.e., chat) AI system built on the GPT architecture, which leverages extensive pre-training on vast textual data to generate coherent, context-aware, and human-like responses tailored for interactive dialogues.

3 https://www.mckinsey.com/featured-insights/mckinsey-explainers/what-is-prompt-engineering.

4 Democratization refers to using LLMs as a means to lower the entry bars to AI/ML tools, making complex workflows such as data organization, traceability, and modeling more accessible and user-friendly to a wider range of researchers and practitioners, thereby enhancing interdisciplinary collaboration and driving progress in science and computation.

5 Artificial General Intelligence (AGI): A form of AI capable of understanding, learning, and performing any intellectual task that a human can, with general adaptability across domain.
